# Electroretinographic effects of retinal dragging and retinal folds in eyes with familial exudative vitreoretinopathy

**DOI:** 10.1038/srep30523

**Published:** 2016-07-26

**Authors:** Yukari Yaguchi, Satoshi Katagiri, Yoko Fukushima, Tadashi Yokoi, Sachiko Nishina, Mineo Kondo, Noriyuki Azuma

**Affiliations:** 1 Department of Ophthalmology and Laboratory for Visual Science, National Center for Child Health and Development, Tokyo, Japan; 2 Department of Ophthalmology, The Jikei University School of Medicine, Tokyo, Japan; 3 Department of Ophthalmology, Osaka University Graduate School of Medicine, Suita, Osaka, Japan; 4 Department of Ophthalmology, Mie University Graduate School of Medicine, Tsu, Japan

## Abstract

We evaluated the retinal function of retinal dragging (R_drag_) and radial retinal folds (R_folds_) in eyes with familial exudative vitreoretinopathy (FEVR) using full-field electroretinography (ERG). Seventeen eyes of nine patients with FEVR who had R_drag_ or R_folds_ were retrospectively studied. Eyes were classified into four groups according to the severity of the retinal alterations: Group 1, without R_drag_ or R_folds_ (5 eyes); Group 2, with R_drag_ (4 eyes); Group 3, with R_folds_ (6 eyes); and Group 4, with R_folds_ in which all major retinal vessels were involved (2 eyes). The amplitudes of all ERG components and the implicit times of the photopic a- and b-waves and 30-Hz flicker responses were decreased or prolonged as the severity of the retinal alterations increased (*P* < 0.01). The photopic negative response was most severely affected and nearly undetectable in all eyes in Groups 3 and 4, although the other ERG components were detectable in all eyes in Group 3 and one eye in Group 4. These results suggest the decrease of retinal functions was correlated with the degree of severity of R_drag_ and R_folds_ in eyes with FEVR. In addition, the function of the retinal ganglion cells appears to be more severely affected compared with the others.

Radial retinal folds (R_folds_) which extend from the optic disc to the peripheral retina are present in fetal and neonatal eyes including eyes with familial exudative vitreoretinopathy (FEVR), retinopathy of prematurity, Bloch-Sulzberger syndrome (incontinentia pigmenti), Norrie disease, and congenital toxoplasmosis^1^,^2^,^3^. Although R_folds_ can resemble the stalk of persistent fetal vasculature, they are different^2^,^4^,^5^,^6^,^7^.

FEVR is a hereditary vitreoretinal abnormality, which is associated with R_folds_ and also with diverse fundus alterations^2^,^6^,^8^. Previous studies of FEVR have confirmed that the fundus appearance progresses from retinal dragging (R_drag_) to R_folds_, and the morphological features of R_drag_ and R_folds_ have been evaluated in detail by ophthalmoscopy, fundus photography, fluorocein angiography (FA), and optical coherence tomography^6^,^8^,^9^,^10^,^11^. However, the retinal function in eyes with R_drag_ or R_folds_ has been investigated by electroretinography (ERG) in only one study^12^.

The retinal function can be evaluated objectively by full-field ERGs qualitatively and quantitatively. Because the neurons giving rise to the different ERG components have been identified, it is possible to determine which type of neuron is altered by a specific disease process^13^. Thus, it should be possible to determine which neural elements are affected by R_drag_ and R_folds_ in eyes with FEVR.

The purpose of this study was to determine which neural elements were affected by R_drag_ and R_folds_ in eyes with FEVR. We also determined whether there was a significant correlation between the severity of the retinal morphological alterations and to the degree of electrophysiological alterations of the different components of the full-field ERGs in eyes with FEVR. To accomplish this, we recorded full-field ERGs from 17 eyes of nine patients with FEVR and analyzed the different components of the scotopic and photopic ERGs.

## Materials and Methods

The protocol for this study was approved by the Institutional Review Board of the National Center for Child Health and Development, and they adhered to the tenets of the Declaration of Helsinki. This study was a retrospective observational case series.

We studied nine patients with FEVR who had R_drag_ or R_folds_ in at least in one eye and had undergone complete ophthalmic examinations under general or local anesthesia. The diagnosis of FEVR, R_drag_, and R_folds_ was made according to the findings reported^2^,^11^. No eyes had other abnormalities that affected the ERG measurements except for FEVR, R_drag_, and/or R_folds_. Our ophthalmic examinations included ophthalmoscopy, fundus photography, fluorescein angiography (FA), and full-field ERGs. Fundus photography and FA were performed with the TRC-50LX (TOPCON, Co. Tokyo, Japan) and RetCam 3 (Clarity Medical Systems, Inc., Pleasanton, CA) cameras.

The full-field ERGs were recorded with a contact lens electrode, and the ERGs were elicited by white light-emitting diodes (LED) built into the contact lens electrode. The stimulus duration, intensity, and the background intensity were controlled by the electrical current delivered to the LEDs by a specially designed LED driver (WLS-20, Mayo Co., Inazawa, Japan)^14^. The stimulus conditions were set according to the guideline of International Society of Clinical Electrophysiology of Vision^13^. The stimulus durations were 0.03 ms for the scotopic rod ERGs and 5.0 ms for the combined rod-cone ERGs. The amplitudes and implicit times of each component of the ERGs were measured as reported previously^13^,^15^,^16^.

The pupil was fully dilated with a combination of topical 0.5% tropicamide and 0.5% phenylephrine hydrochloride. The cornea was anesthetized by topical 0.4% oxybuprocaine hydrochloride before the contact lens electrode was inserted. The reference and ground electrodes were attached to the forehead and earlobe, respectively. The signals were amplified and bandpass filtered between 1 and 300 Hz (Neuropack 8, Nihon Kohden, Tokyo).

For controls, ERGs were recorded from 44 normal eyes under general anesthesia or local anesthesia. The mean ± standard deviation age of the controls was 7.5 ± 8.2 years, and they were one eye from normal eyes and the fellow normal eyes of unilateral peripapillary staphyloma, morning glory syndrome, and optic nerve hypoplasia. The 5 to 95 percentile amplitudes and implicit times for all ERG components of these normal eyes were used as the control values.

**Figure 1:**
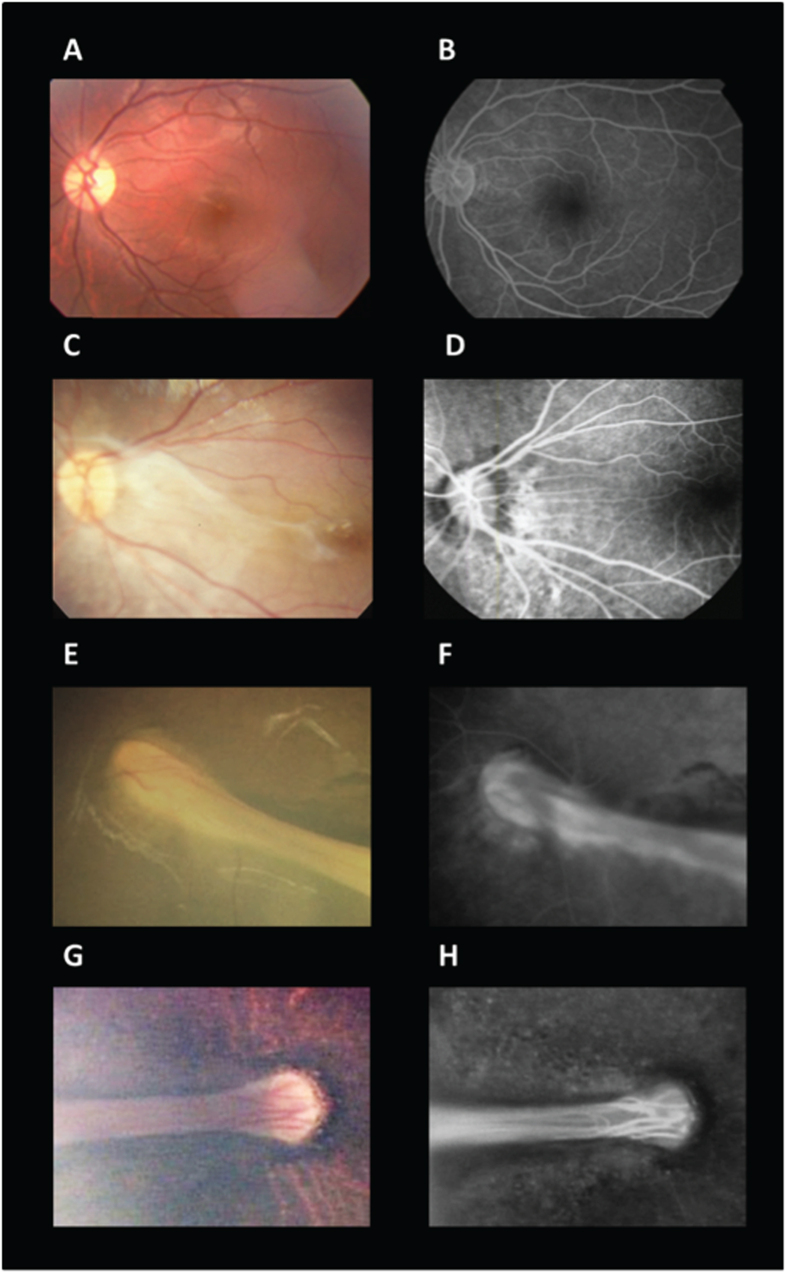
Representative fundus photographs and fluorescent angiograms (FA) of eyes from the four groups; Group 1, without retinal dragging (R_drag_) or retinal folds (R_folds_); Group 2, with R_drag_ only; Group 3, with R_folds_ only; and Group 4, with R_folds_ in which all major retinal vessels were involved. (**A**) The left eye of an 18.0-year-old woman classified in to Group 1 (Case 9). The fundus photograph (Left) and FA (Right) show no straightening of the retinal vessels. (**B**) The left eye of a 15.2-year-old young woman classified in to Group 2 (Case 8). Fundus photograph (Left) and FA (Right) show a straightening of the retinal vessels. (**C**) The left eye of a 1.8-year-old boy classified in to Group 3 (Case 5). Fundus photograph (Left) show radial R_folds_. FA (Right) show residual retinal vessels out of the R_folds_. (**D**) The right eye of a 1.2-year-old girl classified in to Group 4 (Case 2). Fundus photograph (Left) show R_folds_. FA (Right) show no main vessels out of the R_folds_.

The eyes were classified into four groups according to the degree of morphological alterations of the retina: Group 1, without R_drag_ and R_folds_; Group 2, with R_drag_ only; Group 3, with R_folds_ only; and Group 4, with R_folds_ in which all major retinal vessels were affected. Representative fundus photographs and FAs of these four groups are shown in Fig. 1. We defined the eyes in Group 1 as those with an avascular area in the peripheral retina but without straightening of the retinal vessels in the posterior retina or fibrovascular tissue in the periphery. The eyes in Group 2 were those with straightening of the retinal vessels and fibrovascular tissue in the peripheral retina without R_folds_.

The significance of the correlations between the fundus grading (Groups 1 to 4) and the amplitudes and implicit times of each ERG component was determined by Spearman’s rank correlation coefficient.

## Results

Seventeen eyes of nine patients with FEVR were studied. The age at the time of the examinations ranged from 0.7 to 18.0 years (Median age, 10.5 years). One eye with R_folds_ in the nasal retina was excluded. The number of eyes in each group was: Group 1 had five eyes (29.4%); Group 2 had four eyes (23.5%); Group 3 had six eyes (35.3%); and Group 4 had two eyes (11.8%). All of the R_drag_ and R_folds_ existed in the temporal areas of the retina.

**Figure 2:**
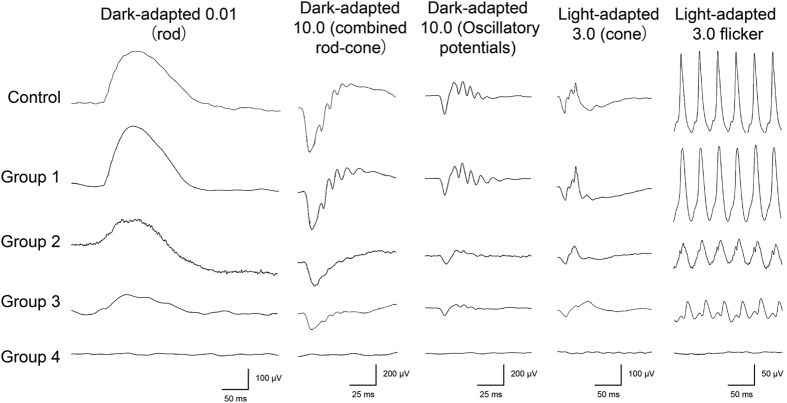
Waveforms of full-field electroretinogram (ERG) obtained from a normal control and representative cases from Groups 1 to 4 of eyes with familial exudative vitreoretinopathy (FEVR). The ERGs of Groups 1 to 4 were obtained from the same eyes shown in Fig. 1.

**Figure 3:**
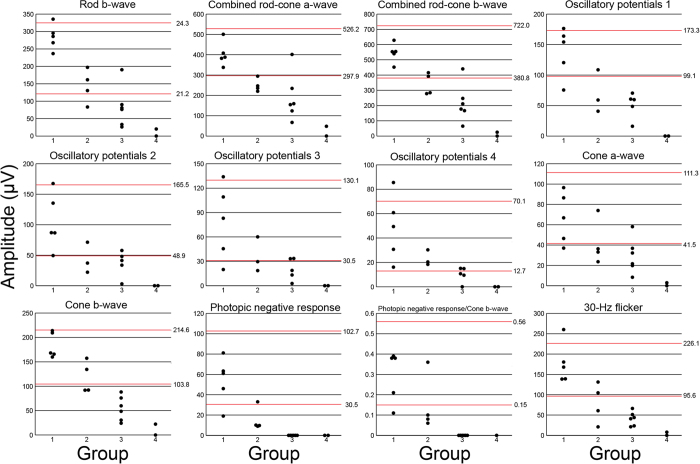
A plot of the average amplitudes of each ERG component for Groups 1 to 4 of the 17 eyes with FEVR. The red upper and lower lines represent the 5 and 95 percentiles obtained from the 44 normal control, respectively. The amplitudes of all ERG components are significantly decreased as the degree of the R_drag_ and R_folds_ progresses (Groups 1–4, *P* < 0.01).

**Figure 4:**
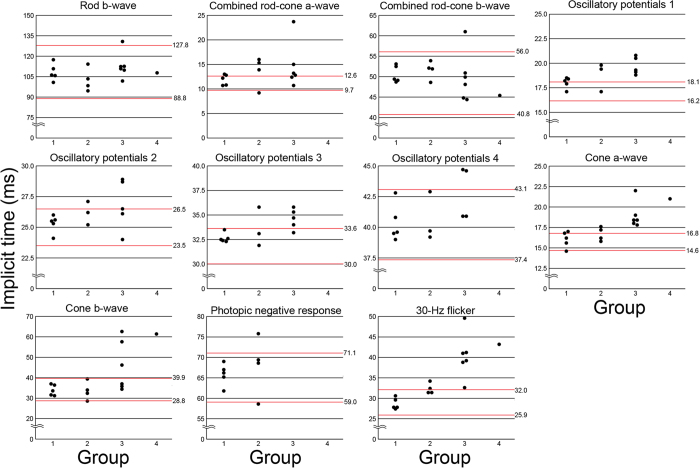
Plot of the implicit times of each ERG component for Groups 1 to 4 of the 17 eyes with FEVR. The red upper and lower lines represent the 5 and 95 percentiles obtained from the 44 normal control, respectively. The implicit times of the cone a- and b-waves are significantly more prolonged as the grades of R_drag_ or R_folds_ become more severe (Groups 1–4, *P* < 0.01), but the correlation was not significant for the implicit times of the other ERG components.

Representative full-field ERGs from a normal control and from each of the groups are shown in Fig. 2. All photopic responses of the eyes in Groups 1 and 2, in which the photopic negative response (PhNR) existed, are shown in supplemental Fig. 1. The amplitudes and implicit times of each ERG component for all 17 eyes are also plotted in Figs 3 and 4, respectively, and the detailed data was summarized in supplemental Table 1. In Group 1, four of five eyes had ERG responses with normal amplitudes and implicit times for all components. In Group 2, some of the eyes had reduced amplitudes or delayed implicit times. In Group 3, the amplitudes were further reduced, and five of six eyes had reduced amplitudes for all ERG components. The implicit times in Group 3 were still within normal limits in about half of the eyes except for the cone a-waves which were prolonged in all eyes. The PhNR was undetectable in all Group 3 eyes. In Group 4, one eye had undetectable ERG responses for all components and another eye had severely reduced and delayed responses for all components.

Statistical analyses showed that the grade of severity from R_drag_ to R_folds_ eyes, i.e., from Group 1 to 4, was significantly correlated with the amplitudes of the b-waves of the rods, a- and b-waves and oscillatory potentials of the combined rod-cone responses, the a- and b-waves, PhNR, and PhNR/b-wave of the cone responses, and the 30-Hz flicker responses (*P* < 0.01). The increase in the severity from R_drag_ to R_folds_ eyes was also significantly correlated with the implicit times of the a- and b-waves of the cone responses, and the 30-Hz flicker responses (*P* < 0.01).

## Discussion

Fundus photography and FA are effective methods of assessing the effects of vascular abnormalities in eyes with FEVR^6^,^8^. A delayed arteriovenous transit time in eyes with R_folds_ was reported especially in the outer areas of the R_folds_ in contrast to the rapid perfusion of the vessels within the R_folds_^8^. The late phase of FA showed that the eyes in Group 4 had no major vessels outside the R_folds_ (Fig. 1D) which might be the end stage of the delayed arteriovenous transit times. In other word, all major retinal vessels in these eyes might be involved in the R_folds_ because of the wide retinal area of the retina affected by the R_folds_. The severely premature retinal vessels in eyes with FEVR are rotated around the optic disc and are markedly dilated.

The results of full-field ERGs showed that the degree of the morphological retinal alterations (Groups 1 to 4), which were determined by fundus and angiographic findings, was significantly correlated with the amplitudes of all ERG components. These results suggest that the depression of retinal function over the entire retina increases as the degree of R_drag_ and R_folds_ progresses. In Group 4, the retinal function determined electroretinographically was nearly undetectable indicating severe retinal dysfunction in the entire retina. These retinal dysfunctions can be caused by morphological retinal abnormalities or abnormal development of the retinal cells during progression of the R_drag_ and R_folds_, such as the movements of the sensory retina in the direction of the R_drag_ or R_folds_ in our recent study^11^.

The degree of the retinal abnormalities (Groups 1 to 4) was significantly correlated with the implicit times of the cone a- and b-waves, but were not correlated with the implicit times of the other ERG components. For example, the implicit times of the rod b-wave and combined rod-cone b-wave fell within the normal range in five of six eyes in Group 3, whereas the implicit times of the cone a-and b-waves were delayed beyond the normal range in more than one-half of the six eyes in Group 3 and the implicit times of 30-Hz flicker responses were delayed in all eyes of Group 3. The exact reason for such different degrees of abnormal implicit times of the different ERG components is unclear, but it might be due to different functional impairments of the rod and cone systems in eyes with FEVR.

One of the interesting findings was found in the PhNRs. Our results showed that the amplitudes of PhNR and the ratio of the PhNR/b-wave were significantly decreased in three of four eyes in Group 2 and the amplitude of the PhNR were completely undetectable in all eyes in Groups 3 and 4, whereas the amplitudes of all other components were still detectable in all eyes of Group 3 and one eye in Group 4 (Fig. 3). Because the PhNR is believed to originate from the neural activities of the retinal ganglion cells and their axons^15^,^16^, these findings suggested that the function of the inner retina might be more severely impaired in eyes with R_drag_ and R_folds_ than the other retinal layers. This agrees with our recent optical coherence tomographic findings that the bundling of retinal nerve fiber occurs in the early stage in eyes with R_drag_, and the main components of the R_folds_ are the retinal nerve fibers^11^.

There current study had some limitations in this study. The number of study eyes were relatively small and the developmental effect depends on the patient age in retinal structure and ERG components needs to be considered.

In conclusion, our electrophysiological findings showed that the amplitudes and implicit times of the ERG components were decreased or prolonged with the severity of the morphological retinal alterations in eyes with FEVR. In addition, the function of the ganglion cells might be more impaired than that of other retinal cells in eyes with R_folds_ which may explain the reduced vision even in eyes with relatively mild R_drag_. Thus, it would be more important to focus on the retinal ganglion cells when treating eyes with R_drag_ and R_folds_.

## Additional Information

**How to cite this article**: Yaguchi, Y. *et al.* Electroretinographic effects of retinal dragging and retinal folds in eyes with familial exudative vitreoretinopathy. *Sci. Rep.* **6**, 30523; doi: 10.1038/srep30523 (2016).

## Supplementary Material

Supplementary Information

Supplementary Information
