# Supplementary material for: Electroretinographic effects of retinal dragging and retinal folds in eyes with familial exudative vitreoretinopathy
Source: Sci Rep. 2016 Jul 26;6:30523. doi: 10.1038/srep30523 (PMC4960584; doi:10.1038/srep30523)
Supplement: Supplementary Information [file srep30523-s1.pdf]

Electroretinographic effects of retinal dragging and retinal folds in eyes with familial exudative vitreoretinopathy

Yukari Yaguchi[1], MD, Satoshi Katagiri[1,2], MD, Yoko Fukushima[3], MD, PhD, Tadashi Yokoi[1], MD, PhD, Sachiko Nishina[1], MD, PhD, Mineo Kondo[4], MD, PhD, Noriyuki Azuma[1], MD, PhD

Supplemental Table 1. The clinical data including full-field electroretinography (FF-ERG) in the current study

Case No	Gender	Examined age	Anesthesia	Right/Left	Group	amplitude of FF-ERG											
						rod b-wave	combined rod-cone a-wave	combined rod-cone b-wave	Oscillatory potentials 1	Oscillatory potentials 2	Oscillatory potentials 3	Oscillatory potentials 4	cone a-wave	cone b-wave	PhNR	PhNR/ cone b-wave	30-Hz flicker
1	male	0.7	General	R	4	20.2	47.9	25.0	0.0	0.0	0.0	0.0	2.9	22.3	0.0	0.00	7.9
				L	3	76.3	234.4	246.8	59.7	57.8	33.5	14.9	21.6	48.6	0.0	0.00	40.9
2	female	1.2	General	R	4	0.0	0.0	0.0	0.0	0.0	0.0	0.0	0.0	0.0	0.0	NM	0.0
				L	3	26.1	66.9	64.8	16.0	3.0	2.7	0.0	19.8	24.2	0.0	0.00	23.4
3	male	10.5	Local	R	3	190.3	401.5	440.5	60.6	41.4	13.2	15.1	58.1	88.2	0.0	0.00	51.2
				L	1	295.4	500.7	539.8	75.5	87.0	19.9	16.1	86.5	209.4	81.1	0.39	167.9
4	female	11.2	Local	R	2	161.5	246.8	391.1	NM	NM	NM	NM	36.3	157.4	9.7	0.06	104.7
				L	3	33.2	123.8	166.5	NM	NM	NM	NM	8.3	30.5	0.0	0.00	21.1
5	male	1.8	General	R	2	197.1	294.6	415.5	108.6	71.3	60.1	30.3	74.0	134.5	10.1	0.08	131.2
				L	3	90.2	159.2	176.9	48.6	33.7	18.8	9.5	36.8	75.9	0.0	0.00	43.9
6	female	1.8	General	R	1	335.5	382.4	553.3	176.4	135.4	109.2	60.8	96.5	214.0	46.0	0.21	260.3
				L	3	80.8	154.5	211.3	70.4	48.0	33.2	10.7	32.2	59.8	0.0	0.00	66.2
7	male	13.2	Local	R	1	236.6	388.0	452.0	154.2	86.7	83.0	49.4	37.0	159.9	61.0	0.38	138.5
				L	2	83.4	220.3	284.3	40.7	37.1	29.6	20.4	23.6	92.2	33.0	0.36	20.9
8	female	15.2	Local	R	1	267.8	337.7	554.6	120.4	49.4	45.5	30.7	46.5	165.8	19.0	0.11	139.1
				L	2	130.5	235.6	278.2	59.1	22.1	18.6	18.4	33.1	91.9	9.0	0.10	60.7
9	female	18.0	General	L	1	285.9	407.9	628.4	163.9	167.6	134.0	85.5	66.8	168.2	63.3	0.38	180.4

Case No	Gender	Examined age	Anesthesia	Right/Left	Group	implicit time of FF-ERG										
						rod b-wave	combined rod-cone a-wave	combined rod-cone b-wave	Oscillatory potentials 1	Oscillatory potentials 2	Oscillatory potentials 3	Oscillatory potentials 4	cone a-wave	cone b-wave	PhNR	30-Hz flicker
1	male	0.7	General	R	4	107.8	NM	45.4	NM	NM	NM	NM	21.0	61.4	NM	43.2
				L	3	109.8	13.2	44.4	19.3	26.1	34.7	44.7	18.4	35.8	NM	32.6
2	female	1.2	General	R	4	NM	NM	NM	NM	NM	NM	NM	NM	NM	NM	NM
				L	3	112.6	23.7	50.9	20.8	28.9	35.3	NM	22.0	62.6	NM	41.2
3	male	10.5	Local	R	3	110.8	12.8	49.9	18.8	24.0	33.2	40.9	17.8	37.0	NM	39.2
				L	1	110.8	12.2	49.4	18.5	24.1	32.3	39.0	15.6	33.6	66.2	27.8
4	female	11.2	Local	R	2	98.4	13.9	53.9	NM	NM	NM	NM	15.8	28.6	58.6	34.2
				L	3	112.4	12.4	61.0	NM	NM	NM	NM	19.0	34.4	NM	49.6
5	male	1.8	General	R	2	94.6	9.2	48.6	17.1	25.2	31.9	39.2	16.2	39.4	68.6	31.4
				L	3	101.8	10.7	48.1	19.1	26.5	34.0	40.9	18.4	57.6	NM	38.8
6	female	1.8	General	R	1	117.4	10.7	49.1	17.1	25.6	32.4	39.6	14.6	37.0	67.0	29.6
				L	3	130.8	15.0	44.8	20.5	28.7	35.8	44.6	18.0	46.2	NM	41.0
7	male	13.2	Local	R	1	106.2	13.0	48.7	18.2	26.0	32.5	39.5	16.2	31.6	65.2	27.8
				L	2	114.2	16.0	51.9	19.8	27.1	33.1	39.7	17.6	32.4	69.4	31.4
8	female	15.2	Local	R	1	100.8	12.8	53.1	18.4	25.5	32.6	40.8	16.8	31.2	61.8	27.4
				L	2	103.4	15.3	52.1	19.4	26.2	35.8	42.9	17.2	34.0	75.8	32.4
9	female	18.0	General	L	1	105.8	10.8	52.5	17.9	25.3	33.5	42.8	17.0	36.4	69.0	30.6

No = number, PhNR = photopic negative response, R = right, L = left, NM = not measured
