# Supplementary material for: Electroretinographic effects of retinal dragging and retinal folds in eyes with familial exudative vitreoretinopathy
Source: Sci Rep. 2016 Jul 26;6:30523. doi: 10.1038/srep30523 (PMC4960584; doi:10.1038/srep30523)
Supplement: Supplementary Information [file srep30523-s2.pdf]

Electroretinographic effects of retinal dragging and retinal folds in eyes with familial exudative vitreoretinopathy

Yukari Yaguchi[1], MD, Satoshi Katagiri[1,2], MD, Yoko Fukushima[3], MD, PhD, Tadashi Yokoi[1], MD, PhD, Sachiko Nishina[1], MD, PhD, Mineo Kondo[4], MD, PhD, Noriyuki Azuma[1], MD, PhD

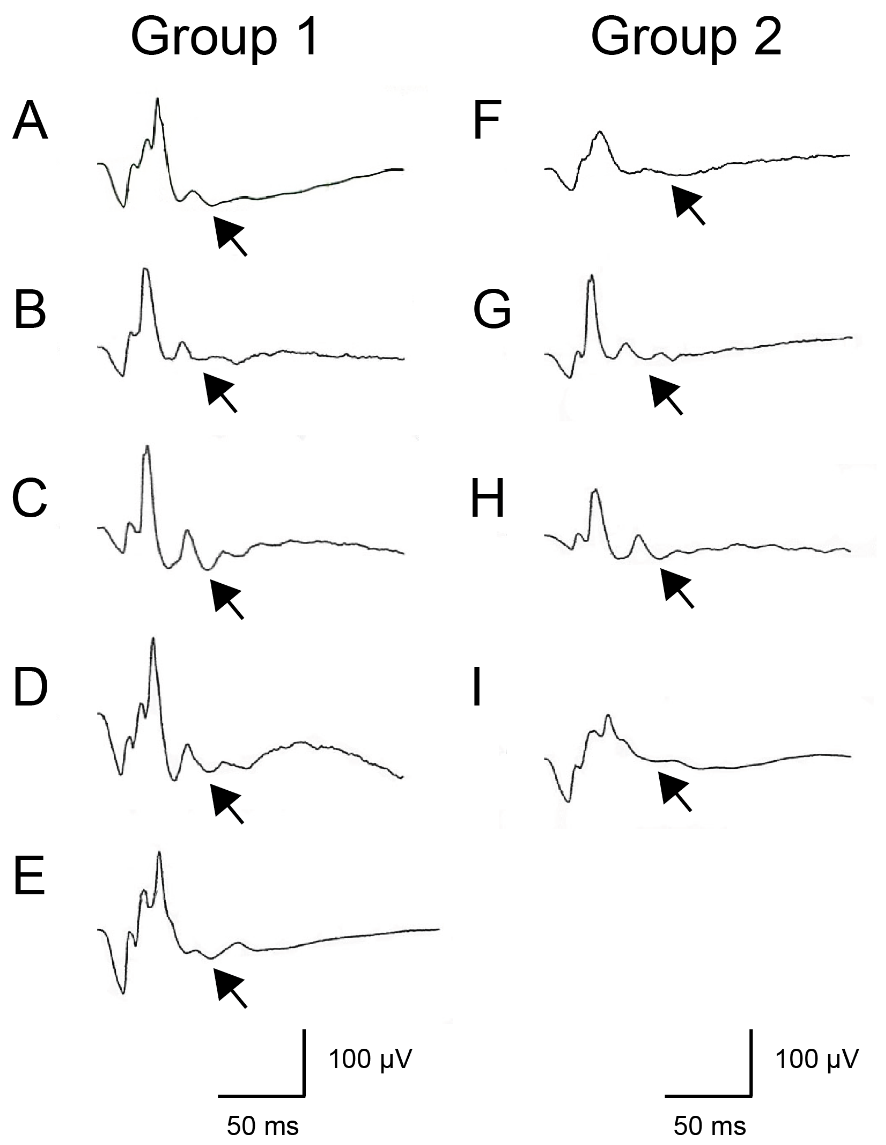

Supplemental Figure 1: The waves of the photopic response in the eyes in Groups 1 and 2 with measureable photopic negative responses. Group 1 (A, B, C, D, and E) and Group 2 (F, G, H, and I). A) The left eye of case 9. B) The right eye of case 8. C) The right eye of case 7. D) The left eye of case 3. E) The right eye of case 6. F) The left eye of case 8. G) The right eye of case 3. H) The left eye of case 7. I) The right eye of case 5. The black arrows indicate the positions of the photopic negative responses in each wave.
